# 

**Published:** 1883-04

**Authors:** 


SURG GENLS OFFS

Vol. XX. April, 1883. No. 1.

### The Bistoury.

Thad S. Up de Graff, M.D.
EDITOR.

ELMIRA, N.Y.

